# Sex- and tissue-specific profiles of chemosensory gene expression in a herbivorous gall-inducing fly (Diptera: Cecidomyiidae)

**DOI:** 10.1186/1471-2164-15-501

**Published:** 2014-06-20

**Authors:** Martin N Andersson, Elin Videvall, Kimberly KO Walden, Marion O Harris, Hugh M Robertson, Christer Löfstedt

**Affiliations:** 1 Department of Biology, Lund University, Lund SE-223 62, Sweden; 2 Department of Entomology, University of Illinois, Urbana-Champaign, IL 61801, USA; 3 Department of Entomology, North Dakota State University, Fargo, ND 58108-6050, USA

**Keywords:** Hessian fly, *Mayetiola destructor*, Gene expression, Genome, Transcriptome, Odorant receptor, Ionotropic receptor, Gustatory receptor, Odorant binding protein, Sensory neuron membrane protein

## Abstract

**Background:**

The chemical senses of insects mediate behaviors that are closely linked to survival and reproduction. The order Diptera contains two model organisms, the vinegar fly *Drosophila melanogaster* and the mosquito *Anopheles gambiae*, whose chemosensory genes have been extensively studied. Representing a third dipteran lineage with an interesting phylogenetic position, and being ecologically distinct by feeding on plants, the Hessian fly (*Mayetiola destructor* Say, Diptera: Cecidomyiidae) genome sequence has recently become available. Among plant-feeding insects, the Hessian fly is unusual in ‘reprogramming’ the plant to create a superior food and in being the target of plant *resistance* genes, a feature shared by plant pathogens. Chemoreception is essential for reproductive success, including detection of sex pheromone and plant-produced chemicals by males and females, respectively.

**Results:**

We identified genes encoding 122 odorant receptors (OR), 28 gustatory receptors (GR), 39 ionotropic receptors (IR), 32 odorant binding proteins, and 7 sensory neuron membrane proteins in the Hessian fly genome. We then mapped Illumina-sequenced transcriptome reads to the genome to explore gene expression in male and female antennae and terminal abdominal segments. Our results reveal that a large number of chemosensory genes have up-regulated expression in the antennae, and the expression is in many cases sex-specific. Sex-specific expression is particularly evident among the *Or* genes, consistent with the sex-divergent olfactory-mediated behaviors of the adults. In addition, the large number of *Ors* in the genome but the reduced set of *Grs* and divergent *Irs* suggest that the short-lived adults rely more on long-range olfaction than on short-range gustation. We also report up-regulated expression of some genes from all chemosensory gene families in the terminal segments of the abdomen, which play important roles in reproduction.

**Conclusions:**

We show that a large number of the chemosensory genes in the Hessian fly genome have sex- and tissue-specific expression profiles. Our findings provide the first insights into the molecular basis of chemoreception in plant-feeding flies, representing an important advance toward a more complete understanding of olfaction in Diptera and its links to ecological specialization.

## Background

Insects comprise the largest group of animals on Earth and most species are heavily dependent on chemoreception for survival and reproduction. Due to ecological adaptations, the gene families involved in chemoreception have evolved, both in terms of gene numbers and sequences, to become highly divergent across different insect taxa [1]. Furthermore, in a particular species, expression of the genes is regulated differently depending on, for instance, sex, tissue, life stage, or physiological state. The sequencing of insect genomes and more recently also of transcriptomes from chemosensory appendages, have paved the way for identification and characterization of the gene families that are important for chemoreception e.g. [1-6].

Several multi-gene families encode proteins with crucial roles in olfaction and taste, including both receptors and non-receptor proteins. Receptors encoded by three large gene families are involved in chemoreception and expressed in sensory neurons, located mainly on the antennae and to a lesser extent on other sensory appendages [7]. These families encode the odorant receptors (OR), gustatory receptors (GR), and ionotropic receptors (IR) [8-10]. The ORs and GRs belong to the same receptor superfamily [11]. While the ORs detect volatile chemicals (odors), the GRs are responsible for contact chemoreception and also for detection of carbon dioxide [7,10]. The IRs constitute a more ancient family of chemoreceptors [8], including both conserved "antennal" IRs involved in olfaction, as well as "divergent" IRs that seem to play a role in taste [12]. The sensory neuron membrane proteins (SNMP) are expressed in pheromone-responding neurons in *Drosophila* and Lepidoptera, and have in some cases been shown to be important for proper pheromone responses [13-15]. Finally, the odorant binding proteins (OBP) are small soluble proteins, highly abundant in the sensillum lymph [16,17]. OBPs bind odor molecules, most of which are hydrophobic, and transport them through the hydrophilic environment in the sensillum to the membrane-bound receptor. OBPs have also been shown to improve the specificity of pheromone-detecting neurons [18,19], but see [20].

Chemosensory gene families of insects have been most extensively studied in two model organisms that both belong to the order Diptera (flies), namely *Drosophila melanogaster*, which feeds on yeast and sugar both as larva and adult, and *Anopheles gambiae*, which feeds on organic material as an aquatic larvae and on blood as an adult female or nectar as a male e.g. [11,21-23]*.* The herbivores are a third major group within the Diptera in which the chemosensory genes so far had not been studied. Here we performed the first analysis of the chemosensory gene families in a herbivorous dipteran species, namely the Hessian fly, *Mayetiola destructor* Say (Diptera: Cecidomyiidae). The large gall midge family Cecidomyiidae (>6000 species) is an example of a group of flies that uses plants as food and contains a large number of important pests of agriculture and forestry [24,25]. Cecidomyiids belong to the suborder Nematocera (the ‘lower’ Diptera) and are phylogenetically interesting because they are positioned between mosquitoes, also in the Nematocera, and *Drosophila*, in the other suborder of the Diptera, the Brachycera [26,27]. Plant-feeding cecidomyiid larvae have highly specialized antagonistic relationships with host plants and have been called ‘reprogrammers of plant genomes’ [28] because of their ability to manipulate the plant to create a specialized nutritive tissue that provides a superior diet for the larva [29,30], a trait that is thought to have contributed to adaptive radiation [31,32]. Most gall midges have a very narrow host range [25]. Other characteristics of cecidomyiids are a very short adult lifespan of 1–2 days or less, during which all reproductive activities must be completed, and mate location by the male is mediated by species-specific female-produced sex pheromones [33]. Cecidomyiids are also special in that adults either do not feed or only rarely feed on water or perhaps nectar [24].

The Hessian fly is a serious pest on wheat (*Triticum* spp.) in many of the world’s major wheat-growing regions [25,34-36]. It is one of the most thoroughly studied plant-feeding insect species, with particular attention on behavioral and sensory ecology, genetics contributing to plant interactions, pheromone communication, pest management, and plant resistance [25,37-42]. The Hessian fly larva has an interaction with plants that is common for plant pathogens but rare for insects, being the target of a ‘gene-for-gene’ plant defense, mediated by *resistance* genes to which it can adapt via mutations in a matching *avirulence* gene [25,43]. Like other gall midges, adults of *M. destructor* have a maximum lifespan of 1–2 days, which limits their behavioral repertoire [25]. Essentially, males emerge from pupation sites in or near the soil and take flight to find virgin females, whereas females emerge with a full complement of mature eggs, release a pheromone to attract a mate, and then continuously search for and lay eggs on a large number of plants until death occurs [37,38,44,45]. Chemical cues are crucial for mate finding, host finding, and oviposition behavior and are, thus, clearly essential for the reproductive success of the Hessian fly.

By having an interesting phylogenetic position between *Drosophila* and mosquitoes, as well as very different behavioral and ecological traits, the Hessian fly is an attractive model for studies of the chemosensory gene families that underlie pheromone detection and host plant finding and assessment. The Hessian fly genome sequence has recently become available ( http://agripestbase.org/hessianfly). Within the framework of the Hessian fly Genomics Consortium, we annotated a total of 228 genes involved in chemoreception (including genes encoding ORs, GRs, IR, SNMPs, and OBPs), corresponding to the first identification of the chemosensory gene families in the Cecidomyiidae. In the present study, we used Illumina RNAseq to sequence transcriptomes of the male and female antennae and terminal abdominal segments, the latter being involved in mating and in the female also in sex pheromone production and host selection behavior. The generated reads were then mapped to the genome sequence to examine the expression profiles of the chemosensory genes in these four tissues. We also analyzed global expression profiles of predicted transcripts and performed gene ontology (GO) annotation. In particular, our results reveal that most of the chemosensory genes have up-regulated expression in the antennae, while a few have up-regulated expression in the terminal abdominal tissues. In addition, a strikingly large number of genes, especially *Or* genes*,* have sex-specific antennal expression, which is likely to reflect the sex-divergent behaviors of adult Hessian flies.

## Results

### Mapping and transcript prediction

We performed Illumina sequencing and subsequently mapped the sequenced reads to the current Hessian fly genome assembly to predict transcripts and analyze gene expression profiles. The sequencing yielded ca. 314 M read-pairs, of which ca. 82 M were derived from the male antennal library, 75 M from female antennae, 77 M from female terminal abdomens, and 79 M from male terminal abdomens. The Illumina reads have been submitted to the Sequence Read Archive (SRA) at NCBI (BioProject accession: SRP041173). The proportion of reads that successfully mapped to the Hessian fly genome assembly was high in all four transcriptomes, ranging from 75% to 80%, despite strict mapping parameters that allowed for only 2 bp mismatches to account for the high sequence similarity among recently duplicated chemosensory genes. The mapping results were used by Cufflinks to predict 27 044 transcripts expressed in female antennae (N50 = 3 211 bp), 24 769 transcripts in male antennae (N50 = 3252 bp), 19 877 in female terminal abdomen (N50 = 3094 bp), and 23 691 in male terminal abdomen (N50 = 2967 bp). Based on these transcripts, 27 029 genes and 36 459 isoforms were predicted. The GC content was 34-35% in all transcriptomes.

### Global transcriptome profiling

#### Gene ontology annotation

GO annotation [46], using Blast2GO ( http://www.blast2go.com/b2ghome) [47,48], was performed to compare the four transcriptomes with regards to the frequency of transcript-associated GO terms. The proportion of transcript sequences with significant BLAST hits was similar in the four transcriptomes, i.e. 57% (15 371 transcripts) for female antennae, 59% (14 539) for male antennae, 65% (13 001) for female terminal abdomen, and 60% (14 265) for male terminal abdomen. The top-hit species distribution (Additional file 1) showed that most hits were represented by sequences from the yellow-fever mosquito (*Aedes aegypti*), followed by the Southern house mosquito (*Culex quinquefasciatus*) and the malaria mosquito (*A. gambiae*). The vinegar fly (*D. melanogaster*) came fourth despite the fact that ca. five times as many protein sequences from *D. melanogaster* have been deposited in the nr database as compared to sequences from *A. aegypti* (Additional file 1). The top BLAST hits for all predicted transcripts as well as their FPKM (Fragments Per Kilobase of transcript per Million mapped reads [49,50]) values and genome locations are given in Additional file 2.

For the female antennae, 41% of the total transcripts had assigned GO terms in addition to BLAST hits. Corresponding numbers in the other transcriptomes were 43% for male antennae, 50% for female terminal abdomen, and 44% for male terminal abdomen. The four transcriptomes turned out to be very similar, both with respect to "Molecular Function" (Additional file 3) and "Biological Process" (Additional file 4) GO annotation. The only notable differences were found in the Molecular Function annotation where the "odorant binding" term only was present in the two antennal tissues, and the "signal receptor activity" term was almost twice as abundant in the two antennal tissues as compared to the two terminal abdominal tissues (Additional file 3). This result is likely to reflect the (chemo)-sensory role of the main sensory organ, the antenna. However, it also indicates that genes coding for proteins with functions (represented by GO-terms) common to all tissue types dominate in this type of analysis that provides no information on the expression levels of the individual genes themselves.

#### Pair-wise transcriptome comparisons

**Figure 1:**
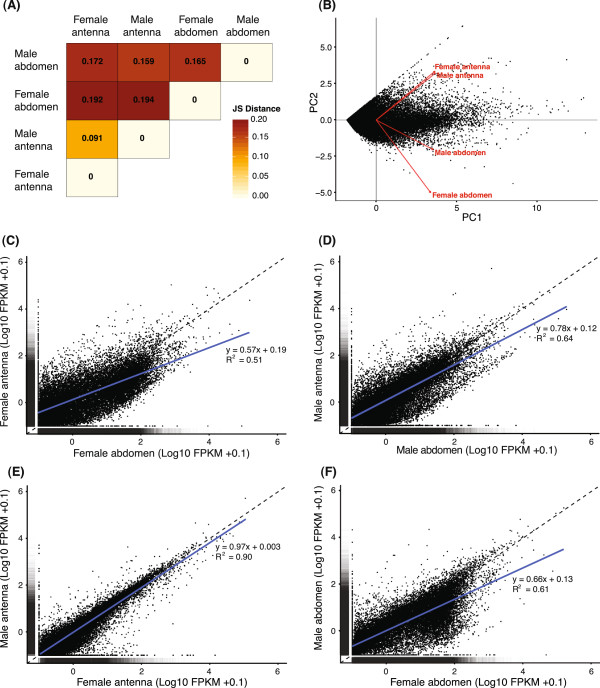
**Comparison of global transcriptome expression profiles.** The overall expression levels of the 27 029 genes predicted from the four transcriptomes were compared using Jensen-Shannon (JS) distance **(A)** and principle component analysis **(B)**, both indicating the high similarity between the male and female antennal transcriptomes. In **(B)**, PC 1 and PC 2 explained 55.8% and 32.2% respectively of the overall gene expression level variation in the four transcriptomes (black dots represent the predicted genes). Both the direction and length (length = within-transcriptome variation) of the eigenvectors (red arrows) show that the male and female antennal transcriptomes had highly similar variation in expression levels, both in relation to PC 1 and PC 2. In contrast, the variation in expression levels differed between the two terminal abdominal transcriptomes, which were also different from the two antennal transcriptomes (as indicated by the different lengths and directions of the eigenvectors). The difference between transcriptomes was explained (mainly) by the expression variation encompassed by PC 2 (i.e. as shown by the position of the four arrow heads). In addition, 1:1 plots (dashed line = 1:1 relationship) in combination with linear regression analyses (blue solid lines) were used to compare transcriptomes pair-wise. The female and male terminal abdomen both had an overall higher expression of the predicted genes compared to the antennae of females **(C)** and males **(D)**, respectively. As indicated by the slope coefficients and relatively low R^2^ values, the expression did not follow a 1:1 relationship in these comparisons. In contrast, expression in the male antennae was similar to that in the female antennae, following a close to 1:1 relationship **(E)**. Finally, the female terminal abdomen had an overall higher expression than the male terminal abdomen, i.e. the relationship between the two transcriptomes was relatively far from 1:1 **(F)**.

The expression levels of the 27 029 genes predicted by Cufflinks were used for global transcriptome profiling. Transcriptomes were compared pair-wise, i.e. male antennae *vs.* female antennae; male terminal abdomen *vs.* female terminal abdomen; male antennae *vs.* male terminal abdomen; and female antennae *vs.* female terminal abdomen. The male and female antennal transcriptomes had very similar global expression profiles, whereas male and female terminal abdomens were more different, both from each other as well as from the two antennal transcriptomes (Figure 1). When divergence based on FPKM values was analyzed using Jensen-Shannon (JS) distance, male and female antennae showed a low level of differentiation (a JS distance of only ca. 0.09). In contrast, the distances in all other pair-wise comparisons were larger, ranging from ca. 0.15 to 0.19 (Figure 1A). The global expression profile similarity between male and female antennae, and their distinctiveness from the other transcriptomes were confirmed in a principle component analysis (Figure 1B), and in pair-wise regression analyses. The latter analyses indicated that the female and male terminal abdominal segments had an overall higher expression than the female and male antennae, respectively (female antennae *vs.* female terminal abdomen: regression slope coefficient = 0.57, R^2^ = 0.51, Figure 1C; male antennae *vs.* male terminal abdomen: slope = 0.78, R^2^ = 0.64, Figure 1D). The male antennae had a similar global expression profile as the female antennae, as shown by a close to 1:1 relationship between the expression levels (slope = 0.97, R^2^ = 0.90, Figure 1E). In general, higher expression was found in the female terminal abdomen as compared to the male terminal abdomen (slope = 0.66, R^2^ = 0.61, Figure 1F).

### Expression of the chemosensory genes

#### Overall expression profiles

Based on our manual annotation of the Hessian fly genome, a total of 228 chemosensory genes from five gene families (*Ors*, *Grs*, *Irs*, *Snmps*, and *Obps*) were predicted. Predicted amino acid sequences of these genes are available in Additional file 5, and phylogenies will be included in the forthcoming publication of the Hessian fly genome (Hessian fly Genomics Consortium, in prep).

**Figure 2:**
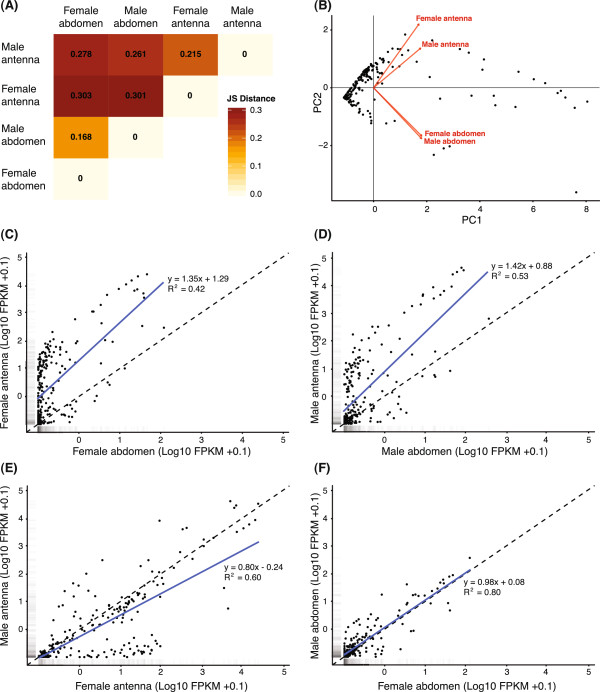
**Comparison of expression profiles of the 228 chemosensory genes.** Expression level profiles of the 228 genes involved in olfaction or taste in the four tissues were compared using **(A)** Jensen-Shannon (JS) distance, and **(B)** principle component analysis. In contrast to the global analysis (Figure 1), the female and male terminal abdomen tissues had the most similar expression profiles, whereas the male and female antennae were more different from each other. In **(B)**, PC 1 and PC 2 explained 66.0% and 20.1% respectively of the overall expression level variation among the chemosensory genes in the four tissues. Both the direction and length (length = within-transcriptome variation) of the eigenvectors (red arrows) indicate that the chemosensory genes (black dots) in the male and female terminal abdominal tissues had highly similar variation in expression levels, both in relation to PC 1 and PC 2. In contrast, the variation in expression levels differed between the male and female antennae, and these tissues were also different from the terminal abdominal tissues (as indicated by differences in length and direction of the eigenvectors). The difference in expression of the chemosensory genes between the four tissues was explained (mainly) by the variation encompassed by PC 2 (i.e. as shown by the position of the four arrow heads). In addition, 1:1 plots (dashed line = 1:1 relationship) in combination with linear regression analyses (blue solid lines) indicate an overall up-regulation of expression of the 228 genes in the antennae of both sexes, compared to the female **(C)** or male terminal abdomen **(D)**, respectively. The antennae of males and females expressed many of these genes differently **(E)**. Overall, the female and male terminal abdomen had low expression of these genes and the expression was closer to a 1:1 relationship than in the other pair-wise comparisons **(F)**.

In contrast to the global comparison where the male and female antennal transcriptomes demonstrated the most similar expression profiles, the female and male terminal abdomens had the most similar profiles when only the 228 chemosensory-related genes were included (Figure 2A, B). Regression analysis demonstrated a close to 1:1 relationship between the expression levels of the chemosensory genes in the two terminal abdominal tissues (regression slope coefficient = 0.98, R^2^ = 0.80, Figure 2F), although the overall expression level was low in these tissues. Most of the chemosensory genes had the highest expression in the female or male antennae as compared to the female or male terminal abdomens (female antennae *vs.* female terminal abdomen: slope = 1.35, R^2^ = 0.42, Figure 2C; male antennae *vs.* male terminal abdomen: slope = 1.42, R^2^ = 0.53, Figure 2D), respectively, although a few genes had higher expression in the terminal abdominal tissues (see below for details). In contrast to the global analysis, the expression profiles of the male and female antennal tissues were clearly different when only the 228 genes related to chemosensation were included in the analysis (slope = 0.80, R^2^ = 0.60, Figure 2E). FPKM values for the chemosensory genes in the four tissues are presented in Additional file 6.

#### Odorant receptors

**Figure 3:**
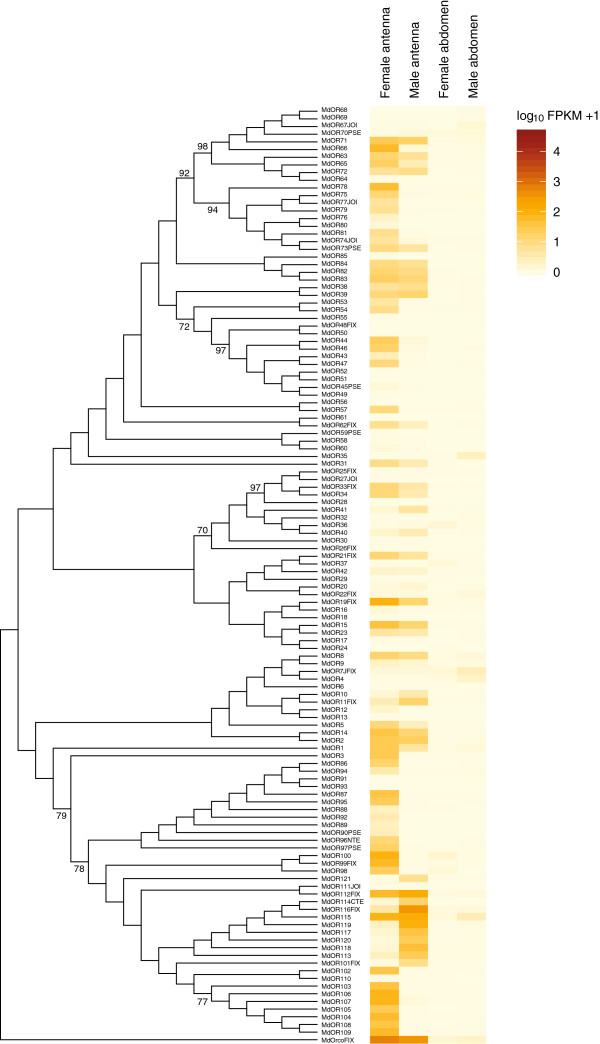
**Phylogenetic relationships of the odorant receptors (OR) and their expression profiles in *M. destructor*.** A distance-corrected neighbor-joining tree (bootstrap consensus, topology only) based on *M. destructor* OR amino acid sequences and rooted by MdORCO. The tree was constructed using Mega 5 after multiple sequence alignment in ClustalX. Numbers on branches indicate bootstrap support (1000 iterations) and are only displayed if >70 and on major branches. Expression levels of the *Or* genes in the four transcriptomes are represented in a heat plot based on log-transformed FPKM values. Zero expression is represented by the lightest yellow color. Suffixes to gene names are explained in the Methods section.

Based on our annotation, the current assembly of the Hessian fly genome contains 122 *Or* genes, including a gene for the conserved co-receptor ORCO and six pseudogenes. Our transcriptome analysis showed that 99 of the *Or* genes were expressed in at least one of the analyzed tissues. Thus, almost 20% (23 genes) of the *Or* genes were not expressed (i.e. genes with less than 10 mapped read-pairs were regarded as having no or biologically insignificant expression). A substantially larger number of *Or* genes were represented by reads in the female antennae (94 *Or* genes) as compared to in the male antennae (64 *Or* genes) (Table 1). Thus, while a large proportion (77%) of the *Or* genes in the genome was expressed in the antennae of the female, only ca. half (52%) of them were expressed in the antennae of the male. Female and male terminal abdomens expressed 24 and 30 *Or* genes, respectively, including *Orco* (Table 1). Thus, relatively large numbers of *Or* genes were expressed outside of the main olfactory organ in both sexes, although the expression level was low in general (Figure 3; Additional file 6). The *Orco* gene had the highest level of expression in both sexes, followed by *Or116* for which the FPKM value in the male antennae was 90% of the FPKM value for *Orco*.

**Table 1:** The number of genes in each chemosensory gene family with detected expression in the four transcriptomes

**Gene family**^**a**^	**Expressed genes**^**b**^			
	**Female antennae**	**Male antennae**	**Female abdomen**	**Male abdomen**
Odorant receptor, OR (122)	94	64	22	30
Gustatory receptor, GR (28)	14	13	7	11
Ionotropic receptor, IR (39)	21	21	11	15
Sensory neuron membrane protein, SNMP (7)	7	7	6	6
Odorant binding protein, OBP (32)	30	31	24	25

^a^Numbers within brackets refer to the number of annotated genes of each chemosensory gene family in the Hessian fly genome.

^b^Expression defined as genes having at least 10 mapped read-pairs.

In the pair-wise comparison between the female antennae and the female terminal abdomen, 85 *Or* genes had higher expression in the antenna (defined by a >3-fold higher FPKM value in combination with a significant chi^2^ – test result, using a Bonferroni-corrected p-value at <2.2 × 10^-4^), whereas two *Or* genes, *Or36* and *Or37*, had higher expression in the terminal abdomen (Figure 3; a list of all the differentially expressed chemosensory genes in the four pair-wise comparisons are presented in Additional file 7). In the corresponding comparison for the male (i.e. antennae *vs.* terminal abdomen), 47 *Or* genes were expressed at a higher level in the antennae, whereas four *Or* genes (*Or4*, *Or7*, *Or35*, and *Or67*) had higher expression in the terminal abdomen. When comparing expression in the antennae of males *vs.* females, 50 *Or* genes showed higher expression in females, whereas 12 were more highly expressed in males. In addition, several of the OR subfamilies in our dendrogram contained receptors of which the majority showed either female or male antennal up-regulation (Figure 3). For instance, the genes for most of the receptors within the clades formed by OR86-97, OR98-100, and OR102-110 were more highly expressed in the female antennae as compared to the male antennae, whereas most of the receptor genes within the clade formed by OR111-121 were more highly expressed in the male antennae. Furthermore, 27 of the *Or* genes showed no expression in the antennae of either males or females, while 33 *Or* genes were similarly expressed in the antennae of the two sexes. Finally, five *Or* genes showed higher level of expression in the female terminal abdomen as compared to the male terminal abdomen, whereas seven *Or* genes had higher expression in the male terminal abdomen in relation to the female terminal abdomen (Figure 3; Additional file 7).

#### Gustatory receptors

**Figure 4:**
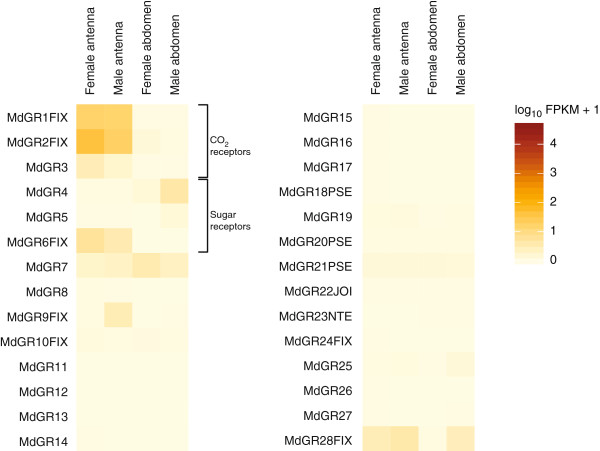
**Expression profiles of the genes coding for gustatory receptors (GR) in *M. destructor*.** Expression levels of the *Gr* genes in the four transcriptomes represented as heat plots based on log-transformed FPKM values. Zero expression is represented by the lightest yellow color. Suffixes to gene names are explained in the Methods section.

We annotated 28 members of the *Gr* gene family in the genome, including genes for the conserved carbon dioxide receptors (*Gr1-3*) [10], three putative sugar receptor genes (*Gr4-6*), and three pseudogenes. However, only 15 of the *Gr* genes were expressed in the tissues analyzed here (Figure 4; Additional file 6). These included the genes for the three putative carbon dioxide receptors and the three sugar receptors. Only slightly larger numbers of *Gr* genes were expressed in the antennae as compared to the terminal abdominal tissues (Table 1). Three of the *Gr* genes (*Gr7*, *Gr10* and *Gr21*) had similar levels of expression in all four tissues (Figure 4).

In the females, six *Gr* genes had up-regulated expression in the antennae as compared to the terminal abdomen. These included the genes for the carbon dioxide receptors (*Gr1-3*) that had the highest expression of all the *Gr* genes, and the putative sugar receptor gene *Gr6*. In contrast, another putative sugar receptor gene, *Gr4*, had up-regulated expression in the female terminal abdomen as compared to the female antennae (Figure 4; Additional file 7). Similar to the females, *Gr1-3* and *Gr6* had higher expression in the male antennae as compared to the male terminal abdomen, whereas *Gr4* had higher expression in the terminal abdomen (Figure 4; Additional file 7). In contrast to the female, the expression of *Gr9* was also up-regulated in the male antennae as compared to terminal abdomen, whereas *Gr28* (up-regulated in the female antennae) was expressed at a similar level in the male antennae and terminal abdomen. *Gr9* was also the only *Gr* gene that was differentially expressed between the two antennal transcriptomes. Finally, four *Gr* genes, including the putative sugar receptor genes *Gr4-5* were more highly expressed in the male terminal abdomen than in the female terminal abdomen. None of the *Gr* genes were more highly expressed in the female terminal abdomen than in the male terminal abdomen.

#### Ionotropic receptors

In total, 39 *Ir* genes, including four pseudogenes were annotated in the Hessian fly genome. Of these, 16 genes encoded "antennal" IRs and 23 encoded "divergent" IRs. We found Hessian fly orthologues for most of the conserved antennal *Ir* genes identified in other Diptera [12], including *Ir8a*, *Ir21a*, *Ir25a*, *Ir41a*, *Ir60a*, *Ir64a*, *Ir68a*, *Ir76b*, *Ir93a*, and seven members of the *Ir75* group. However, *Ir31a*, *Ir40a*, and *Ir92a*, which are found in other Diptera, were not present in the Hessian fly genome and could also not be identified in our transcriptome data. The remaining 23 *MdIr* genes were regarded as members of the divergent class of insect *Irs*.

**Figure 5:**
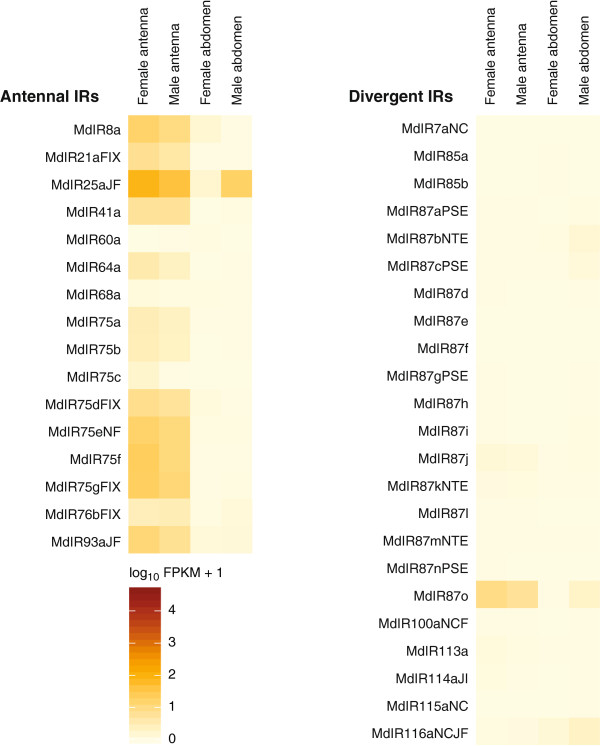
**Expression profiles of the genes coding for ionotropic receptors (IR) in *M. destructor*.** Expression levels of the *Ir* genes in the four transcriptomes represented as heat plots based on log-transformed FPKM values. Zero expression is represented by the lightest yellow color. Suffixes to gene names are explained in the Methods section.

Twelve of the identified *Ir* genes (all divergent) were not expressed, whereas all the antennal *Ir* genes showed expression in at least one of the analyzed tissues (Figure 5; Additional file 6). Larger numbers of *Ir* genes were expressed in the antennal tissues as compared to the terminal abdominal tissues (Table 1). The gene for the broadly occurring co-receptor IR25a had the highest expression of all the *MdIr* genes. Expression of this gene was high in the antennae of both sexes and in the male terminal abdomen.

Expression of 18 *Ir* genes was higher in the female antennae as compared to the female terminal abdomen. Of these genes, 14 encoded antennal IRs and four encoded divergent IRs (Figure 5; Additional file 7). Although IR60a is classified as an "antennal" IR, its gene showed a non-significant tendency for higher expression in the female terminal abdomen than in the female antennae. Apart from that, only one of the divergent *Ir* genes (*Ir116a*) was significantly up-regulated in the female terminal abdomen. In the males, 14 *Ir* genes (13 antennal and 1 divergent) had higher expression in the antennae as compared to the terminal abdomen, while three *Ir* genes had higher expression in the terminal abdomen. Similar to the females, *Ir116a* was one of them, but also *Ir87b* and *Ir87c*.

Only one of the *Ir* genes, *Ir75c*, had up-regulated expression in the female antennae compared to those of the male, representing the only example of differential *Ir* gene expression between the antennae of the two sexes. Comparing the two terminal abdominal tissues, five *Ir* genes, including both antennal and divergent receptor genes, had higher expression in the male terminal abdomen, whereas expression of two *Ir* genes was higher in the female terminal abdomen (Figure 5; Additional file 7).

#### Sensory neuron membrane proteins

**Figure 6:**
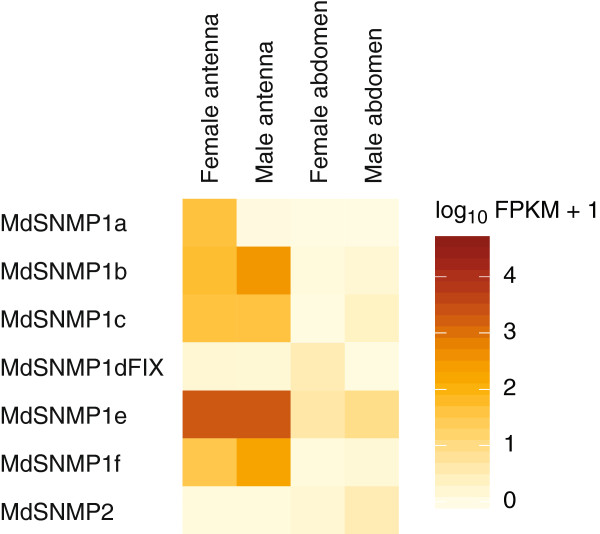
**Expression profiles of the genes coding for sensory neuron membrane proteins (SNMP) in *M. destructor*.** Expression levels of the *Snmp* genes in the four transcriptomes represented as heat plots based on log-transformed FPKM values. Zero expression is represented by the lightest yellow color. Suffixes to gene names are explained in the Methods section.

The Hessian fly genome assembly contained 6 orthologues of *Snmp1* and one *Snmp2*. All of them were expressed in the antennae, and six of them (all but *Snmp1a*) were expressed in both terminal abdominal tissues (Table 1; Figure 6; Additional file 6). In general, the *Snmp* genes had high expression, and the most highly expressed gene (*Snmp1e*) had a higher FPKM value than *Orco*.

In the females, *Snmp1a-c*, *e* and *f* had up-regulated expression in the antennae as compared to the terminal abdomen, while one (*Snmp1d*) was up-regulated in the terminal abdomen, both in relation to the female antennae and to the male terminal abdomen (Additional file 7). Furthermore, *Snmp2* had slightly higher expression in the female terminal abdomen than in the female antennae, but the difference was not significant. The highest expression of *Snmp2* was found in the male terminal abdomen. In the males, *Snmp1b-f* had up-regulated expression in the antennae as compared to the terminal abdomen. Comparing expression of *Snmp* genes between the antennae of the two sexes, *Snmp1a* was greatly up-regulated (260X) in the female. In contrast, *Snmp1b* and *1f* had male-biased antennal expression, while *Snmp1b*, *1c*, and *1e* were similarly expressed in the antennae of the two sexes (Figure 6).

#### Odorant binding proteins

We annotated 32 *Obp* genes in the Hessian fly genome assembly, including two pseudogenes. Generally, OBPs are classified into different phylogenetic groups depending on the number of conserved cysteine residues. Classic OBPs have six cysteine residues, whereas members of the Plus-C class have additional cysteines and one characteristic proline [16,51]. Five of the Hessian fly OBPs (OBP8-12) belonged to the Plus-C class.

**Figure 7:**
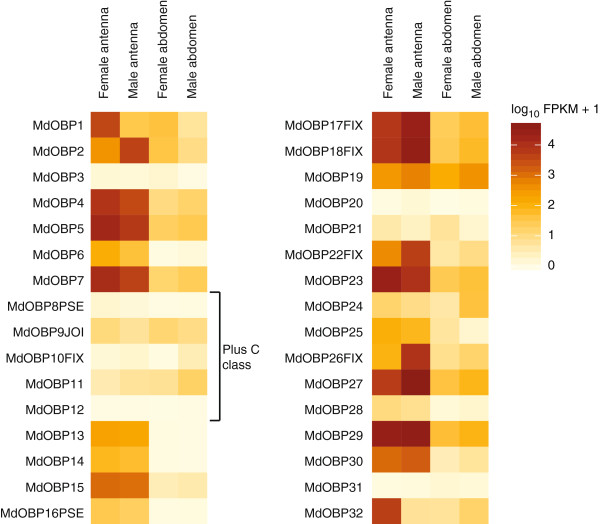
**Expression profiles of the genes coding for odorant binding proteins (OBP) in *M. destructor*.** Expression levels of the *Obp* genes in the four transcriptomes represented as heat plots based on log-transformed FPKM values. Zero expression is represented by the lightest yellow color. Suffixes to gene names are explained in the Methods section.

All but one (*Obp12*) of the 32 *MdObp* genes showed expression in at least one of the analyzed tissues (Figure 7; Additional file 6). Essentially all *Obp* genes were expressed in the antennae of both sexes, while slightly lower numbers of genes were expressed in the terminal abdominal tissues (Table 1). Overall, the most highly expressed *Obp* genes had much higher expression levels (2–3 orders of magnitude) than the most highly expressed *Or* or *Snmp* genes (Additional file 6). The Plus-C class of *Obp* genes (i.e. *Obp8-12*) had comparatively low expression levels in all four tissues (Figure 7).

In the female, most (23) of the *Obp* genes were more highly expressed in the antennae as compared to the terminal abdomen. Only one *Obp* gene (*Obp31*) showed a higher level of expression in the female terminal abdomen than in the female antennae (Additional file 7), but the overall expression level of this gene was comparatively low. The males were similar to the females by having 20 *Obp* genes with higher expression in the antennae as compared to the terminal abdomen. These included most of the genes that also were antennally up-regulated in the female. Three *Obp* genes (*Obp11*, *Obp24* and *Obp32*) had higher expression in the male terminal abdomen as compared to the male antennae. However, despite the fact that most *Obp* genes had their highest expression in the two antennal tissues, their expression level in the terminal abdomen tissues was in general much higher than the expression of the other chemosensory gene families in the abdominal tissues (Additional file 6).

A few *Obp* genes showed differential expression between the male and female antennae (Figure 7; Additional file 7). Only two *Obp* genes (*Obp1* and *Opb32*) showed higher levels of expression in the female antennae, whereas six genes (*Obp2*, *Opb17*-*18*, *Opb22*, and *Opb26-27*) had higher expression in the male antennae. Nine of the *Obp* genes showed differential expression levels between the two terminal abdominal tissues; five of them (*Obp1*-*3, Opb21*, and *Opb25*) were more highly expressed in the female, while four (*Obp10*-*11*, *Opb24*, and *Opb32*) were more highly expressed in the male.

## Discussion

### Chemosensory genes in the Hessian fly in relation to *Drosophila* and mosquitoes

The chemosensory gene families have been extensively studied in various *Drosophila* and mosquito species [11,21,52-56], but had not been analyzed in plant-feeding dipterans, such as gall midges. The Hessian fly in the Cecidomyiidae family is both phylogenetically [26,27] and ecologically [25] well separated from flies and mosquitoes. The specific habits and highly evolved relationship to its host also makes studies of the Hessian fly chemosensory genes interesting for a more complete understanding of olfaction in Diptera. The current Hessian fly genome assembly contains genes for 122 ORs, 28 GRs, 39 IRs, 7 SNMPs, and 32 OBPs. In comparison to *Drosophila* and mosquitoes, the number of Hessian fly *Or* genes is relatively large, whereas the number of genes encoding taste receptors (GRs and divergent IRs) is small [11,21,52,53], suggesting that olfaction is the dominant chemical sense in this species. This might be related to the short adult lifespan, with olfaction being a more time-efficient modality for host and mate assessment, occurring during flight at a distance rather than after landing via contact chemoreception. While clear orthologues of ORs are rarely found in distantly related species, the antennal IRs appear conserved across insect species [12]. However, the Hessian fly genome lacked genes for three antennal IRs (*Ir31a*, *Ir40a*, and *Ir92a*) that are present in other Diptera, suggesting that also some olfactory functions have been lost in this herbivorous species. Insects generally have two *Snmp* genes (*Snmp1* and *Snmp2*), but some species have multiple paralogues of *Snmp1*[57]. SNMP1 has been shown to be important for pheromone detection in *Drosophila*[13]. The Hessian fly genome contains six *Snmp1* genes, which is the largest number identified in any insect so far. It is possible that this large number reflects the use of a multi-component sex pheromone in the Hessian fly [37], and that the detection of the individual pheromone components requires specific OR-SNMP1 combinations.

### Sex-specific antennal expression of chemosensory genes

Transcriptome profiling using RNAseq data has been shown to accurately predict gene expression levels [58], and has recently been used to analyze the expression of chemosensory genes in several other insect species, including *D. melanogaster* and the *A. gambiae* mosquito [6,23,56,59,60]. Here, we used a transcriptome profiling approach to analyze sex- and tissue specific expression in the Hessian fly. Our global transcriptome analysis that included all the ca. 27 000 predicted genes indicated that the gene expression profiles of the male and female antennae were highly similar to each other, while expression profiles of the antennae were different from the terminal abdominal tissues. In addition, the GO annotation also indicated very similar frequencies of transcript-associated GO terms in the two antennal transcriptomes. In fact, the results from the latter analysis were also similar to previous results from GO annotation of antennal transcriptomes of two bark beetle species and the moth *Manduca sexta*[2,3], suggesting that "transcript frequencies" in terms of presence/absence are conserved across insect orders.

When only the 228 chemosensory genes were included in the analysis, the expression profile differed widely between the four tissues. The majority of the chemosensory genes had up-regulated expression levels in the antennae as compared to the terminal abdominal tissues. Furthermore and in contrast to the global analysis, a clear difference in gene expression between the antennae of males and females was found. Although all analyzed chemosensory gene families contained some genes with expression levels that differed between the sexes, the most striking difference was found among the *Or* genes, for which ca. 62% of the genes with any expression showed at least a three-fold significant difference between male and female antennae, and ca. 50% of them showed at least a ten-fold difference, with the majority being female-biased. Thus, the proportion of *Or* genes with sex-biased expression was substantially larger than previously found in *D. melanogaster*[60]*,* a species in which both sexes feed (and females oviposit) on the same food, but similar to *A. gambiae*[6], where males and females feed on entirely different food. Similar to the mosquito, the sex-biased expression in the Hessian fly is likely to reflect the different ecological interests of males and females [37,45,61]. In both sexes, the gene for the co-receptor ORCO [62] had the highest expression levels of all *Or* genes. This is consistent with its broad expression and role in forming heteromeric complexes with the conventional ORs, required for the odor response of the neuron and localization of the OR in the cell membrane [63-66].

During their ca. 18 h adult existence, male Hessian flies do just one thing, namely search for and mate with virgin females, carrying enough sperm to fertilize up to 35 females [67]. Finding females is accomplished by long-range attraction to a female-produced sex pheromone, comprised of five to seven compounds [37,68]. Similar to moths e.g. [69-71], it is likely that the subfamily formed by the receptors with male-biased expression in the Hessian fly (i.e. OR112-121; Figure 3) includes the receptors for the pheromone compounds. We currently test this hypothesis using heterologous assays. Interestingly, two of the *MdOr* genes (*Or112* and *Or115*) in the clade with putative pheromone receptors had high expression levels also in the females, and other receptors in this clade had moderate female expression. Thus, if these ORs are the pheromone receptors, it is likely that females also are able to detect some of their pheromone compounds.

In contrast to the males, for which there is no evidence in cecidomyiids of attraction to host plants [72], adult females depend on plant chemicals to find and select host plants for their offspring [61,73]. Females, during their ca. 9–24 h existence, crawl from an eclosion site in the soil to a perch where they extend the abdominal segments to release a sex pheromone and, after mating, fly to find host plants for their eggs. Attraction to host plants is mediated by an array of volatile chemicals [45,73] and discrimination between host and non-host plants would presumably require a larger and different set of ORs as compared to detection of the pheromone. Thus, it is likely that the 50 *Or* genes with female-biased expression are involved in host plant recognition. Similar to the Hessian fly, most of the *Ors* with sex-biased expression in *A. gambiae* were up-regulated in the female, i.e. the sex that blood-feeds and finds sites for oviposition [6].

Interestingly, the *Or* gene expression profiles in the Hessian fly antennae were often correlated to the phylogenetic position of the ORs in the tree, i.e. the majority of the receptors within a phylogenetic subfamily showed male- or, in most cases, female-biased antennal expression. It is possible that ORs with similar sequences detect structurally similar chemicals. Thus, this result might also reflect the relative importance of host odors for females versus the pheromone compounds for males. A correlation between sex-specific expression bias and position in the phylogeny was also found for the ORs in a recent study on two ant species [59]. This is likely to represent adaptations that enable individuals to carry out the tasks that are specific to their sex or caste.

The majority of the conserved antennal *MdIr* genes had relatively high expression in the antennae, but only one of them (*Ir75c*) showed a clear difference in expression level between the sexes. This suggests that in contrast to the ORs, receptors in this more ancient family [12] detect compounds that might be of general importance for insects, regardless of sex. Of the antennal *Ir* genes, *MdIr60a* was not expressed in the antenna. Similarly, Benton et al. [8] did not detect expression of *Ir60a* in antennal samples of *D. melanogaster*. To our knowledge, a ligand for this receptor has not been identified yet in any species.

The carbon dioxide receptor genes *MdGr1-3* had high expression in the antennae (in both sexes), suggesting that this organ is responsible for carbon dioxide detection, which is similar to *Drosophila* (although *Drosophila* uses an heterodimer receptor, lacking the GR2 subunit) [74], but different from mosquitoes that detect this gas with the maxillary palps [75,76]. Only one *Gr* gene, *MdGr9*, had male antennal-biased expression. In a phylogenetic analysis (Hessian fly Genomics Consortium, in prep), this receptor (together with MdGR7-8) grouped together with DmGR43a, which has been shown to monitor fructose levels in the fly brain [77]. The reason why a tentative fructose receptor would have high expression in the antenna of male Hessian fly is not clear, but this receptor could possibly be important if males do feed on nectar [24].

Since SNMP1 is important for pheromone detection in *D. melanogaster*[13], we expected to find the highest expression levels of the *MdSnmp1* genes in the male antennae of the Hessian fly. However, only two (*Snmp1b* and *1f*) of the six *Snmp1* orthologues present in the Hessian fly genome had higher expression in the male antennae as compared to the female antennae. These two *Snmps* were also relatively highly expressed in the female antennae. Interestingly, *Snmp1a* had >200-fold higher expression in the female antennae as compared to the male antennae. Expression of *Snmp* genes in females might, again, suggest that females also are able to detect components of the sex pheromone, or alternatively, that the role of SNMP1 might not be restricted to detection of sex pheromones. Expression of *Snmp1* in the antennae of both sexes has also been found in other dipteran and non-dipteran species [13,14,78].

In general, the *MdObp* genes had the highest expression levels of all analyzed chemosensory gene families, and the majority of them were most highly expressed in the antennae. Only a few *Obp* genes were differentially expressed between the antennae of males and females, which is similar to *A. gambiae* and the yellow fever mosquito, *Aedes aegypti*[6,79]. Similar to the other gene families, the *MdObp* genes that did show sex-biased expression might be important for the detection of compounds that elicit sex-specific behaviors.

### Expression in terminal abdominal tissues

All chemosensory gene families in the Hessian fly contained several genes that were represented by reads also in the terminal abdominal samples. The *Obp* gene family was the most broadly expressed, followed by the *Snmps*, while the *Or* gene family had the most antennal-specific expression. Nevertheless, all gene families contained genes that were significantly more highly expressed in the male or female terminal abdominal segments as compared to the antennae. In the male, these included four *Ors*, two *Grs*, three *Irs*, one *Snmp*, and three *Obps.* In the female, two *Or* genes and one gene from each of the other families were more highly expressed in the terminal abdomen than in the antennae. These observations suggest that chemosensation is not restricted to the antennae of gall midges. Female Hessian flies examine the leaf surface with the tip of the abdomen prior to oviposition [80], and oviposition is induced by chemical cues from host plants [61,73,81], consistent with the expression of chemoreceptors in this tissue. Similarly, the gustatory sense of *D. melanogaster* in not restricted to head appendages and taste sensilla are present on the legs, wings, and female genitalia (in association with the ovipositor) [7,82]. In addition, *Or59b* in *D. melanogaster* is predominantly expressed in the male accessory gland ( http://flyatlas.org/atlas.cgi?name=CG3569-RA[83]), and a recent study showed that several *Or* genes are expressed in the testes of *A. gambiae* and that ORCO is involved in chemical-induced sperm activation [84]. These results are consistent with the expression of several *MdOrs* including *MdOrco* in the male terminal abdominal tissue analyzed here.

Similar to the present study, other studies have reported that *Snmp1* and *Snmp2* as well as the *Obps* can be expressed in variety of tissues in addition to the chemosensory organs, suggesting that these proteins can have physiological roles independent of olfaction [2,13,14,79,85,86]. The *Snmps* and *Obps* that were expressed in the female Hessian fly terminal abdominal segments where the pheromone gland is found [87], might be involved in the binding and transport of pheromone molecules before these are volatilized and released.

The GR and divergent IR families contained the largest proportions of genes for which we did not detect expression in the present study. Similarly, the five genes (*MdObp8-12*) of the Plus-C class of OBPs showed only low levels of expression. It is possible that these genes are expressed mainly in legs or wings, or in yet other tissues [88], which we did not analyze. Indeed, several *Ir* genes and Plus-C *Obp* genes showed high expression in the labella and tarsi of *A. aegypti*[79]. Alternatively, these and the other "non-expressed" chemosensory genes might be expressed only in the larvae [89-91], which are likely to use contact chemical cues in order to locate a suitable feeding site inside the wheat plant [29].

## Conclusions

By conducting a comprehensive manual gene annotation and transcriptome analysis we found clear sex- and tissue-specific expression patterns of the chemosensory genes in the Hessian fly. While many studies of these genes have been performed on Diptera, especially *Drosophila* and mosquitoes, this is the first of a fly that feeds on plants. The expression profile difference between male and female Hessian fly was more pronounced than that observed in *D. melanogaster*, but similar to *A. gambiae*, a species that also displays sex-divergent chemically-mediated behaviors. We found the most extreme sex-specific expression difference among the *Or* genes, which is likely to reflect the sex-divergent olfactory behaviors of the short-lived adults. In addition, the relatively large number of *Or* genes in the genome in combination with the few *Gr* genes and divergent *Ir* genes, suggest that long-range attraction to hosts and mates is more important than contact assessment in this species, in which the adults have reduced mouthparts and feeding behavior has not been documented. We also found that several genes of each gene family had expression that was not restricted to the antennae, indicating that they serve multiple and tissue-specific physiological roles. Future studies should focus on characterizing the function of the proteins encoded by these differentially expressed chemosensory genes in order to consolidate the link between the genetics and ecology of Hessian fly olfaction.

## Methods

### Identification of chemosensory genes in the genome

Full details of our manual annotation protocol for the genes encoding the ORs, GRs, IRs, SNMPs, and OBPs in the Hessian fly genome will be included in the publication of the genome (Hessian fly Genomics Consortium, in prep). In brief, reciprocal tBLASTn searches against the *M. destructor* genome sequence (assembly 1.0, available at the Agricultural Pest Genomics Resource Database: http://www.agripestbase.org) were conducted to identify the chemosensory genes*.* Removal of introns and identification of start and stop codons were performed manually, aided by a multiple sequence alignment and a Trinity *de novo* transcriptome assembly based on our Illumina reads. Genes only partially assembled in the genome were corrected/extended manually and suffixes added to the name of such genes: "FIX" was added to manually extended genes; "JOI" to joined fragments of the same gene (with support from transcriptome data), but assembled on two or three scaffolds; "NTE" and "CTE" to genes with missing N or C terminus, respectively; "PSE" to pseudogenes; and "INT" to genes with internal exons missing. One-letter abbreviations were used for genes with multiple suffixes, (i.e. F, J, N, C, and I). These suffixes are used also in the present study.

### Insects and RNA isolation

The Hessian fly was reared using methods described in [92]. Strains of Hessian fly differ in their ability to live on wheat genotypes with resistance mediated by *H* genes [93]. We used the ‘Great Plains’ biotype, which is avirulent (i.e. dies) on any wheat genotype that carries an *H* gene. It is also the same biotype that was used for genome sequencing. Females laid eggs on 1–2 leaf seedlings of susceptible ‘Roblin’ spring wheat (*Triticum aestivum* L.) planted in large pots (20 cm diam.) in a greenhouse (21-24°C, 60-85% RH). Puparia were removed from the plant and put on moist soil in Plexiglas cages under controlled environmental conditions (20°C, 70% relative humidity, 16:8 h light:dark photoperiod) for the adults to emerge.

Male and female antennae, as well as the 8^th^-10^th^ female and male terminal abdominal segments, the latter including ovipositor and pheromone gland tissue in the females [87], and the clasper in the males, were excised from newly emerged adults and put in separate tubes held at -80°C. In total, body parts from 283 males (566 antennae) and 320 females (640 antennae) were collected and used for RNA isolation. Tissues were homogenized using a rotor-stator homogenizer (Tissue-tearor model 985370–395, Biospec Products Inc., USA), and total RNA extracted using the RNeasy Mini kit (Qiagen), following the manufacturer’s instructions. The extraction yielded 3.5 μg total RNA from male antennae, 2.4 μg from female antennae, 6.5 μg from female terminal abdomen, and 5.5 μg from male terminal abdomen.

### Illumina sequencing

The RNAseq libraries were prepared from cDNA averaging 230 nt using Illumina’s TruSeq RNAseq Sample Prep kit. The four transcriptome libraries were pooled in equimolar concentration, quantified by qPCR, and sequenced from both ends of the fragment (100 bp reads) using a TruSeq SBS sequencing kit (version 3). Two lanes were sequenced on a HiSeq2000 at the University of Illinois, Urbana-Champaign, Il, USA. The data were analyzed with Casava 1.8 (pipeline 1.8) [94], which also filtered out low-quality reads. Adaptor sequences were also removed from the dataset. The sequencing yielded ca. 314 M read-pairs from the four transcriptomes and for the two lanes in total.

### Read mapping, transcript and gene prediction

The genome sequence (assembly 1.0) of *M. destructor* was downloaded from http://agripestbase.org/hessianfly/?q=download. Subsequently, the Illumina reads from the four transcriptomes were mapped separately to the genome assembly using TopHat (v. 2.0.8, with parameters for shorter introns) [95], allowing for two base-pair mismatches. Strict mapping criteria were used in order to account for the high sequence similarity among several of the recently duplicated chemosensory genes (mainly *Ors*). Cufflinks (v. 2.1.1) [50] with multi-mapped read correction was then used to predict transcripts based on the mapping results, and estimation of gene expression levels in FPKM values (Fragments Per Kilobase of transcript per Million mapped reads). Cuffmerge and Cuffdiff [49,50] were then used for prediction of genes by merging the predicted transcripts in the four tissues, allowing for comparison of FPKM values in the four transcriptomes at each predicted gene locus.

### Gene ontology annotation

For an assessment of the transcript populations predicted by Cufflinks in the four tissues, we performed gene ontology (GO) annotation with Blast2GO ( http://www.blast2go.com/b2ghome) [47,48]. Blast2GO uses hierarchical vocabularies to assign genes or transcripts GO terms related to Biological Process, Cellular Component or Molecular Function, allowing for meta-analysis of gene populations [46]. In the GO annotation, we included all procedures recommended to achieve the highest accuracy and most comprehensive GO annotation [48], all of which are provided in the Blast2GO software [47]. The initial BLASTx step was performed with an E-value cut-off at 10^-3^, the mapping step using default settings, and the annotation step with an E-value cut-off at 10^-3^, and default settings for both GO-weight (5) and annotation cut-off (55). Subsequently, to increase the number of annotated transcripts, all transcripts with BLAST hits were included in InterProScan searches at the EBI (including the following applications: BlastProDom, FPrintScan, HMMPIR, HMMPfam, HMMSmart, HMMTigr, ProfileScan, HAMAP, PatternScan, SuperFamily, SignalPHMM, TMHMM, HMMPanther, Gene3D, Phobius, and Coils [48,96]). These results were then merged to the existing GO annotations via the "merge" step in Blast2GO (as described in [48]). The annotations were then further augmented using the ANNEX procedure, which verifies and adds "related" GO terms to transcripts that already have assigned GO terms, and the subsequent Blast2GO validation step (for details see [48]). In the present study, merging the InterProScan results to the GO annotation increased the number of annotated transcripts by 9–11% in the four transcriptomes, whereas the ANNEX step increased the number of GO terms with 7–8%.

### Expression level analysis

The Cufflinks-predicted transcripts and genes and their FPKM values were used for whole transcriptome profiling. However, some of the chemosensory gene transcripts were not accurately predicted by Cufflinks, probably due the high sequence similarity of tandemly duplicated genes. Therefore, HTSeq (v. 0.5.3p9) [97] and subsequently DESeq (v. 1.12.0) [98] were used for more accurate expression estimations of the chemosensory genes, for which the exact genome coordinates were known. Thus, expression levels (in FPKM values) of the chemosensory genes are based on the number of paired reads mapped to the genome from the first nucleotide of the start codon to the third nucleotide of the stop codon of each gene.

Differences in expression levels for each of the chemosensory genes were analyzed pair-wise between the four transcriptomes using chi^2^ tests that were based on weighted read-pair counts i.e. the number of mapped read-pairs for each gene in relation to the total number of mapped read-pairs in each transcriptome. The following comparisons were made: (i) female antennae *vs.* male antennae, (ii) female antennae *vs.* female terminal abdomen, (iii) male antennae *vs.* male terminal abdomen, and (iv) female terminal abdomen *vs.* male terminal abdomen. We used strict criteria for claiming differential expression; a gene was regarded as being differentially expressed (or "up-regulated" in relation to its expression in the other transcriptome in the pair-wise comparison) only if its FPKM value differed by at least 3-fold between the two analyzed transcriptomes, in combination with a significant Bonferroni-corrected p-value at <2.2 × 10^-4^. Genes with less than 10 mapped read-pairs in both of the transcriptomes under comparison were not analyzed for differential expression, since we considered expression of these genes too low for accurate estimates. Instead, these genes were considered as having no or biologically insignificant expression.

## Availability of supporting data

All data supporting the results of this article are included in the additional files. The read sequences from the four transcriptomes have been submitted to the Sequence Read Archive (SRA) at NCBI under accession number SRP041173 and can be accessed at http://www.ncbi.nlm.nih.gov/sra/?term=SRP041173. The phylogenetic tree (ORs) and underlying alignment file have been deposited at LabArchives ( http://www.labarchives.com): DOI 10.6070/H4251G56.

## Abbreviations

FPKM: Fragments per kilobase of transcript per million mapped reads; GO: Gene ontology; GR: Gustatory receptor; IR: Ionotropic receptor; OBP: Odorant binding protein; OR: Odorant receptor; SNMP: Sensory neuron membrane protein.

## Competing interests

The authors declare that they have no competing interests.

## Authors' contributions

MNA, HMR, and CL initiated the project, conceived and coordinated the study. MNA, KKOW, and HMR performed gene annotation and bioinformatics. MNA performed RNA isolation, gene ontology analysis, and drafted the manuscript. EV performed read-mapping, transcript prediction, and gene expression analysis. KKOW carried out transcriptome assemblies. MOH reared the insects, provided biological material and contributed substantially to manuscript preparation. MNA, EV, KKOW, HMR, and CL analyzed and interpreted the data. All authors contributed to study design and manuscript preparation. All authors have read and approved the final manuscript.

## Supplementary Material

Additional file 1 **Top BLAST-hit species distribution for all predicted transcripts in the four transcriptomes.** Hits for 15 371 transcripts from female antennae, 14 539 transcripts from male antennae, 13 001 transcripts from female terminal abdomen, and 14 265 transcripts from male terminal abdomen. Click here for file

Additional file 2 **Genome locations, FPKM values and top BLAST hits for all transcripts predicted by Cufflinks.** Included are data for 27 044 predicted transcripts from female antennae, 24 769 transcripts from male antennae, 19 877 transcripts from female terminal abdomen, and 23 691 from male terminal abdomen. Click here for file

Additional file 3 **Results from Gene Ontology (GO) Molecular Function annotation.** Representation of transcript sequences based on frequency of associated Molecular Function (MF) GO terms at level 3 GO categorization for: **(A)** 11 015 annotated transcripts in female antennae (22 318 MF terms in total), and **(B)** 9 922 transcripts in female terminal abdomen (22 664 MF terms). The annotation was done separately for each of the four transcriptomes, but since the GO results were essentially identical in the two sexes, only the female results are shown for simplicity. GO terms relevant for chemosensation that differed in abundance between the antennae and terminal abdomen are underlined and highlighted in bold. Click here for file

Additional file 4 **Results from Gene Ontology (GO) Biological Process annotation.** Representation of transcript sequences based on frequency of associated Biological Process (BP) GO terms at level 2 GO categorization for: **(A)** 11 015 GO annotated transcripts in female antennae (38 314 BP terms in total), and **(B)** 9 922 transcripts in female terminal abdomen (43 892 BP terms). The annotation was done separately for each of the four transcriptomes, but since the GO results were essentially identical in the two sexes, only the female results are shown for simplicity. Click here for file

Additional file 5 **Predicted amino acid sequences of the chemosensory genes.** Included are odorant receptors, gustatory receptors, ionotropic receptors, sensory neuron membrane proteins, and odorant binding proteins identified in the Hessian fly genome. Click here for file

Additional file 6 **FPKM values for the 228 chemosensory genes in the four transcriptomes.** Included are values for 122 odorant receptors (OR), 28 gustatory receptors (GR), 39 ionotropic receptors (IR), 7 sensory neuron membrane proteins (SNMP), and 32 odorant binding proteins (OBP). Suffixes to gene names are explained in the Methods section. Click here for file

Additional file 7 **Lists of chemosensory genes differentially expressed among the four transcriptomes.** Expression levels in the following tissues were compared: female antennae *vs.* female terminal abdomen, male antennae *vs.* male terminal abdomen, female antennae *vs.* male antennae, and female terminal abdomen *vs.* male terminal abdomen. Differential expression was defined by at least a 3-fold difference in the FPKM value of a gene in a comparison between two transcriptomes in addition to a significant Bonferroni-corrected p-value at <2.2 × 10^-4^ after a chi^2^ test. Suffixes to gene names are explained in the Methods section. Click here for file
